# Prevalence and risk factors for diabetes and diabetic retinopathy: results from the Nigeria national blindness and visual impairment survey

**DOI:** 10.1186/1471-2458-14-1299

**Published:** 2014-12-18

**Authors:** Fatima Kyari, Abubakar Tafida, Selvaraj Sivasubramaniam, Gudlavalleti VS Murthy, Tunde Peto, Clare E Gilbert, The Nigeria National Blindness and Visual Impairment Study Group

**Affiliations:** International Centre for Eye Health, Department of Clinical Research, London School of Hygiene and Tropical Medicine, London, UK; Department of Ophthalmology, College of Health Sciences, University of Abuja, Abuja, Nigeria; Ministry of Health, Dutse, Jigawa State Nigeria; Medical Statistics Team, Division of Applied Health Sciences, University of Aberdeen, Aberdeen, UK; Indian Institute of Public Health, Public Health Foundation of India, Hyderabad, Andra Pradesh India; Moorfields Eye Hospital, London, UK; NIHR Biomedical Research Centre at Moorfields Eye Hospital and UCL Institute of Ophthalmology, London, UK

## Abstract

**Background:**

In Nigeria, urbanisation and increasing life expectancy are likely to increase the incidence of non-communicable diseases. As the epidemic of diabetes matures, visual loss from diabetic retinopathy (DR) will increase unless mechanisms for early detection and treatment improve, and health systems respond to the growing burden of non-communicable diseases.

**Methods:**

A nationally-representative population-based sample of 13,591 participants aged ≥40 years selected by multistage-stratified-cluster-random-sampling with probability-proportional-to-size procedures were examined in 305 clusters in Nigeria between January 2005 to June 2007. All were asked about history of diabetes and underwent basic eye examination. Visual acuity (VA) was measured using logMAR E-chart. Participants with VA<6/12 and/or DR detected underwent detailed eye examination including dilated retinal examination and retinal photography. Systematic sampling of 1-in-7 gave a subsample (n=1759) examined in detail regardless of VA; and had random blood glucose (RBG) testing. Images were graded by Moorfields Eye Hospital Reading Centre. Participants were defined as having diabetes if they were previously diagnosed or RBG>11.1mmol/l or had DR. Data in the subsample were used to estimate the prevalence and to analyse risk factors for diabetes and DR using multivariable logistic regression. Additional information on the types of DR was obtained from participants not in the subsample.

**Results:**

In the subsample, 164 participants were excluded due to missing data; and 1,595 analysed. 52/1,595 had diabetes, a prevalence of 3.3% (95%CI 2.5-4.3%); and 25/52(48%) did not know. Media opacity in 8/52 precluded retinal examination. 9/44(20.5%) had DR. Higher prevalence of diabetes was associated with urban residence (Odds ratio [OR]1.87) and overweight/obesity (OR3.02/4.43 respectively). Although not statistically significant, DR was associated with hypertension (OR3.49) and RBG>15.0mmol/L (OR8.10). Persons with diabetes had 3 times greater odds of blindness. Of 11,832 other participants in the study sample, 175(1.5%) had history of diabetes; 28 had DR. Types of DR (total=37) included 10.8% proliferative, 51.4% macular oedema.

**Conclusion:**

The age-adjusted prevalence of diabetes in Nigeria was 3.25% (95%CI 2.50-4.30) and over 10% of people with diabetes aged ≥40 years had sight-threatening-DR. These data will enable the development of better public health strategies for the control of diabetes and planning services for DR to prevent vision loss.

## Background

The number of people (aged 20-79 years) with diabetes mellitus (diabetes) worldwide is projected to increase from 382 million in 2013 to 592 million in 2035 [1]. India and other parts of Asia will have the highest number of people with diabetes by 2035, but the highest percentage increase will be in the Middle Eastern Crescent (+96%) and Sub-Saharan Africa (+109%) [1]. In Sub-Saharan Africa the number of people with diabetes is projected to increase from 19.8 million in 2013 to 41.4 million in 2035 [1] but public health strategies for managing the emerging diabetes epidemic are inadequate or non-existent.

Globally, diabetic retinopathy (DR) accounts for 5% of all blindness, affecting 2 million people [2], and it is the leading cause of blindness in people aged 15 – 64 years in industrialized countries. Diabetic retinopathy can be classified into two broad categories: non-proliferative DR (NPDR) and proliferative DR (PDR). PDR and diabetic macular edema (DME) are both sight-threatening and can result in visual impairment and/or blindness. The major risk factors for DR are long duration of diabetes, poor glycaemic control and hypertension [3], and there is evidence from clinical trials that early treatment of PDR and DME can preserve visual acuity [4]. Visual loss from DR is, therefore, potentially avoidable. Indeed, it has been estimated that blindness from DR could be reduced by as much as 90% if agreed treatment protocols and standardized care for diabetics were to be implemented [2].

In Nigeria, a national survey of non-communicable diseases undertaken in 1992 reported the national prevalence of diabetes to be 2.8% (95% CI 2.6-3.1%) in persons aged 15 years and above [5]. In another survey in an urban population in southern Nigeria the prevalence of diabetes was 6.8% (95% CI 4.6-9.0%) among those aged 40 years and above [6], while other studies reported prevalence figures ranging from 1.6% to 12.7% in those aged 15 years and above [7–12]. In Ghana the adjusted prevalence of diabetes was 6.4% (95% CIs not reported) among those aged 25-years and above [13]. However, none of the studies in West Africa reported the proportion of persons with diabetes who had DR.

In Nigeria there is rapid urbanisation and increasing life expectancy, so an increase in the incidence of non-communicable diseases (NCD), including diabetes, is to be anticipated. Indeed, data from the Nigeria national blindness and visual impairment survey showed that the prevalence of hypertension is already very high (44.9%; 95% CI 43.5-46.3%) [14].

Nigeria has the largest population of all African countries, being 128 million at the time of the national blindness and visual impairment survey (January 2005 to June 2007). Nigeria has six main administrative divisions/geopolitical zones (GPZ), 36 states and a Federal Capital Territory; 50.3% of the population live in urban areas, and 62.6% live below the poverty line [15] despite a rapidly increasing GDP.

This paper reports findings in relation to diabetes and DR from the Nigeria national blindness and visual impairment survey, which involved participants aged 40 years and above across the country between 2005 and 2007. The following are presented in this paper: the prevalence of diabetes and risk factors for diabetes; the prevalence and types of DR and risk factors for DR, and the causes of vision loss in participants with diabetes. The national survey gave a prevalence estimate for blindness (presenting visual acuity [VA] of <3/60 in the better eye) of 4.2% (95% CI 3.8-4.6%) [16]. Cataract was the commonest cause of blindness (43%) and DR was responsible for 0.5% of blindness [17]. More in-depth data on diabetes and DR will enable the development of better public health strategies for the control of diabetes, and planning services for DR to prevent vision loss.

## Methods

### Study design and clinical assessment

**Figure 1:**
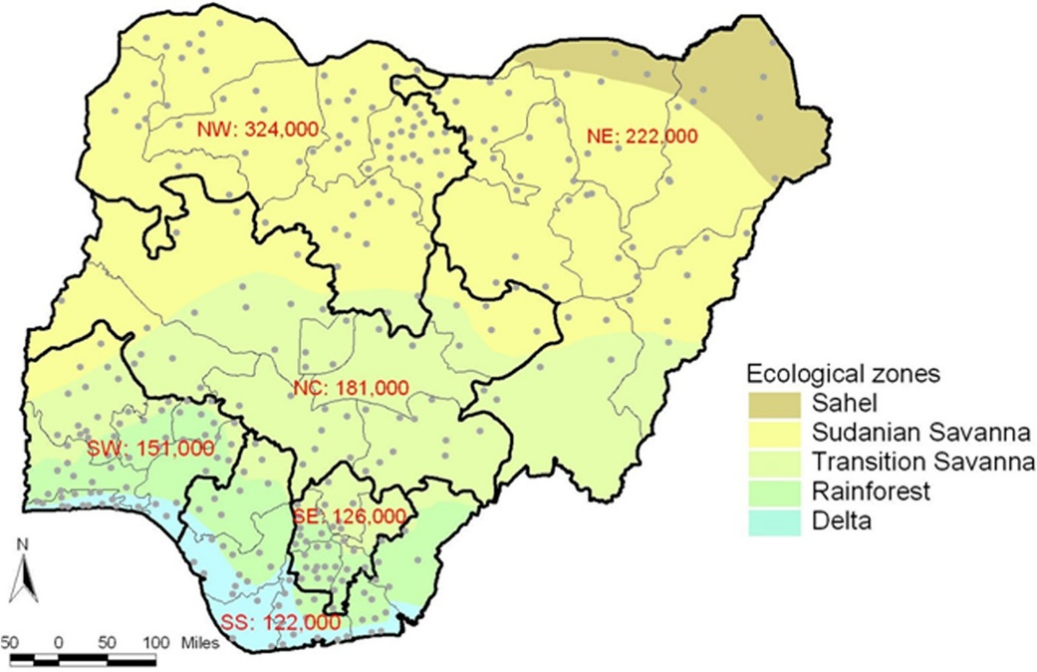
**Map of Nigeria showing cluster sites and magnitude of blindness for the Nigeria National Blindness and Visual Impairment Survey.**

The Nigeria national blindness and visual impairment survey was a cross-sectional population-based survey designed to determine the prevalence and causes of blindness and visual impairment. Multistage stratified cluster random sampling with probability-proportional-to-size procedures were used to ensure a nationally representative sample, and 13,591 of the 15,027 enumerated individuals aged 40 years and above were examined (response rate 90%) in 305 clusters across the 36 states and Federal Capital Territory of Nigeria (Figure 1). Survey methods have been described in detail, including, quality assurance and data management [18]. Data were collected by two teams of Nigerians each comprising two ophthalmologists, one optometrist and two ophthalmic nurses. Inter-observer agreement studies of visual acuity (VA) testing were performed during pilot studies undertaken in each of the six geopolitical zones (GPZ), which are the main administrative divisions in the country. The average kappa statistic for VA testing were 0.53 (moderate agreement) for inter-observer differences of one or less letters; and 0.39 (fair agreement) for inter-observer differences of two or less letters. For Mehra-Minassian lens opacity grading [19] the kappa was 0.70 (substantial agreement).

The study adhered to the tenets of the Declaration of Helsinki and ethical approval was obtained from the Ethics Committee of the London School of Hygiene & Tropical Medicine and the Federal Ministry of Health, Nigeria. Informed verbal consent was obtained from community leaders, heads of households and all participants. All participants identified with ocular pathology requiring assessment and/or treatment were referred to the nearest eye service, including those with sight threatening DR (STDR).

### Selection of the subsample

**Figure 2:**
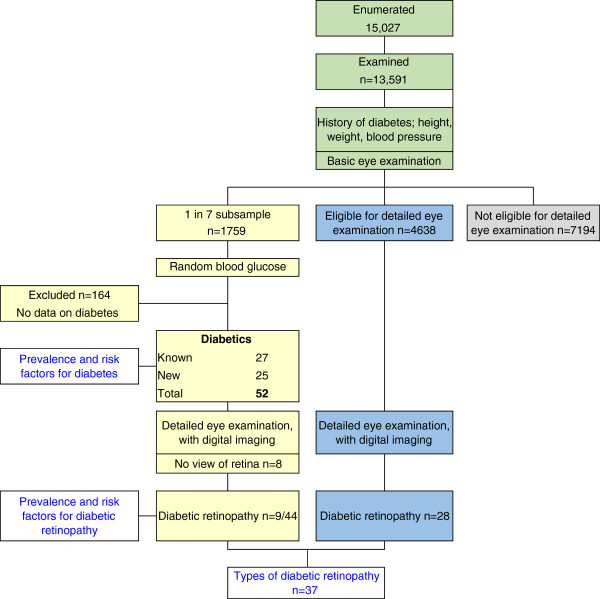
**Flow chart showing how different denominators were derived for analyses of diabetes and diabetic retinopathy.**

Systematic sampling of 1-in-7 of all participants (N = 13,591) at the time of registration gave a subsample (n = 1,759) (Figure 2), who were all examined in detail (see below) regardless of presenting VA or ocular findings. Participants in the subsample had random blood glucose (RBG) testing of capillary blood drawn with a lancet finger-prick (Omron one-touch ultra blood glucose meter). This subsample was used to estimate the prevalence of diabetes and DR, and to assess their risk factors.

### Procedures on all participants

All participants were interviewed to obtain information on personal and demographic details and they were asked whether they had diabetes previously diagnosed by a doctor or were on treatment for diabetes. Height was measured to the nearest tenth of a centimeter and weight to the nearest 100gram using standard equipment. Blood pressure (BP) was recorded three times with BP Omron wrist instrument (Omron Healthcare Ltd, Milton Keynes, England) after resting for at least 10 minutes. Average values were used in this analysis. Presenting and best corrected VA were measured using a reduced logMAR tumbling-E chart. [20, 21] All participants had a basic eye examination by an ophthalmologist.

### Detailed eye examination

All participants in the subsample as well as participants with a VA <6/12 in one or both eyes and/or DR or other posterior ocular pathology seen on undilated fundoscopy also underwent detailed eye examination. Detailed examination was performed by experienced ophthalmologists and included slit-lamp biomicroscopy (Zeiss SL 115 Classic Slit Lamp, Carl Zeiss Meditec AG Jena Germany) and dilated retinal examination using 60D aspheric condensing lens (Volk) and binocular indirect ophthalmoscopy (BIO; Keeler all-pupil) with a 20D lens. Lens opacities were graded using the Mehra-Minassian [19] and World Health Organization (WHO) grading systems [22]. Participants also had digital retinal photography (Zeiss Visucam Lite Desk Top Fundus Camera, Carl Zeiss Meditec AG Jena Germany) focused on the optic nerve head and the macular region through a dilated pupil. Images were graded by Moorfields Eye Hospital Reading Centre (MEHRC).

### Data on the types of diabetic retinopathy

Data on the types of DR were obtained from two sources. First, from diabetics identified in the subsample and second from the larger number of participants not in the subsample (*n* = 11,832) (Figure 2) in whom DR was detected.

### Definition of diabetes, diabetic retinopathy and vision loss

Diabetes was defined as a self-reported positive history of diabetes, or a RBG of 11.1 mmol/l or higher [23] (subsample only) and/or DR was detected on dilated retinal examination and/or was identified by MEHRC from images. Among those with a history of diabetes, the duration of diabetes was not ascertained as this was likely to be very unreliable and it was anticipated that a high proportion of diabetics would not have been diagnosed. In the MEHRC, images were viewed “full screen” on a 24-inch Eizo S2433W monitor calibrated using a DataColor Spyder 2 calibrator or a 24-inch widescreen Dell 2407wfp monitor calibrated using a Gretag Macbeth Eye One Display 2 calibrator. Diabetic retinopathy was defined as the presence of microaneurysms, dot-blot haemorrhages, intra-retinal microvascular anomalies (IRMA), new vessels on the disc or elsewhere, cotton-wool spots, exudates and clinically significant macular edema. Diabetic retinopathy was classified as NPDR, PDR and DME based on a modified ETDRS classification [24]. If retinal images were not available nor readable due to media opacity, findings recorded by the examining ophthalmologists were used.

Visual acuity was classified using WHO categories which use the presenting VA in the better eye. Moderate visual impairment (VI) was defined as <6/18 to >6/60; severe VI as <6/60 to >3/60, and blindness as <3/60 [25]. An additional category of mild VI was included i.e. <6/12 to >6/18. All participants with a presenting VA of <6/12 in one or both eyes were examined in detail, and all possible causes of vision loss were listed for each eye. The most preventable or treatable disorder was then selected as the main cause for the person, using WHO guidelines [26].

### Data analysis and statistical methods

- Subsample (n = 1,759)
  Participants with a positive history of diabetes were classified as known diabetics, those with a raised RBG who were unaware they had diabetes were classified as new diabetics, and those with missing data on their diabetes status were excluded. This dataset was used to estimate the prevalence of diabetes and DR, and for analyses of their risk factors (n = 1,595).
  The risk of diabetes and DR were assessed in relation to socio-demographic factors (increasing age, gender, ethnicity and literacy); location (urban residence and GPZ) and biophysical factors (hypertension and body mass index). Risk factors for DR also included axial length. The association of diabetes with vision loss was also assessed. Age was grouped in 10-year categories; ethnic groups with >100 participants (Fulani, Hausa, Ibo and Yoruba) were analysed separately, combining smaller ethnic groups into an “others” category. Literacy was defined by the ability to read and write and urban residence was defined as a settlement of more than 20,000 people. Hypertension was defined as WHO stage 1 for systolic/diastolic BP of >140/90 mmHg, stage 2 > 160/100 mmHg and stage 3 > 180/110 mmHg [27]. Body mass index (BMI) was calculated by dividing body weight (kg) by height (m) squared and categorized according to the WHO international classification for adults i.e., underweight (<18.5 kg/m^2^), normal (18.5–24.9 kg/m^2^), overweight (25.0–29.9 kg/m^2^) and obese (>30.0 kg/m^2^) [28]. Random blood glucose was grouped as normal (<11.1 mmol/L), high RBG > 11.1-14.9 mmol/L and high RBG > 15.0 mmol/L. Axial length was assessed as a continuous variable and as quartiles.
  Associations with potential risk factors were explored using the Pearson design-based F test for binary traits and other categorical data. Univariate and multivariate logistic regression analyses were performed to identify significant associations. Risk factors identified in univariate analyses with p-values <0.2 were included in the multivariate analyses. Adjusted odds ratios (OR) with 95% confidence intervals (CI) were calculated. All analyses took account of additional variation introduced by the stratified cluster sampling design. P-values <0.05 were considered as statistically significant.
- Whole dataset
  The number of participants with DR among those not in the subsample (n = 11,832) was also determined. The types of DR and the main cause of vision loss seen in all persons with DR in the whole dataset were also described.
  Missing values were excluded from all analyses. All the analyses were performed using Stata (Stata/IC 13.1; Stata Corp, College Station, TX).

## Results

The subsample was representative of the whole study sample by age, gender and place of residence (Table 1). The mean age (standard deviation [SD]) for the whole sample was 55.9 (12.4) years and for the 1-in-7 subsample was 56.1 (12.1) years. The difference in the means was not statistically significant (p = 0.62). In the subsample, 164 participants had insufficient data to determine their diabetes status and were excluded from the diabetes analysis. The excluded participants did not differ to those analysed with respect to age (p = 0.09), gender (p = 0.74) and place of residence (p = 0.70). The mean age (SD) for those analysed in the subsample was 56.1 (12.0) years and for those excluded in subsample because of missing data was 55.4 (13.3) years. The difference in the means was not statistically significant (p = 0.48).

**Table 1:** **Socio-demographic characteristics of the subsample compared with the whole study population**

	Whole study population N = 13591		1-in-7 subsample n = 1759	
	N	% (95% CI)	n	% (95% CI)
**Socio**-**demographic factors**				
Age-group in years (p = 0.62)				
40-49	4889	36.0 (34.8-37.2)	616	35.0 (32.7-37.4)
50-59	3577	26.3 (25.5-27.2)	461	26.2 (24.1-28.4)
60-69	2773	20.4 (19.6-21.2)	368	20.9 (19.1-22.8)
70-79	1653	12.2 (11.5-12.9)	229	13.0 (11.5-14.7)
80+	699	5.1 (4.7-5.7)	85	4.9 (4.0-5.9)
Gender (p = 0.44)				
Female	7345	54.0 (53.1-55.0)	937	53.3 (51.0-55.5)
Male	6246	46.0 (45.0-46.9)	822	46.7 (44.5-46.8)
Place of residence (p = 0.13)				
Rural	10540	77.6 (72.5-81.9)	1371	77.9 (72.9-82.3)
Urban	3051	22.4 (18.1-27.6)	388	22.1 (17.7-27.1)
**Total**	**13591**	**100**	**1759**	**100**

### Prevalence of diabetes and risk factors associated with diabetes

In the subsample, 164 (9.3%) participants had missing data on diabetes status and so were excluded, leaving 1,595 for analysis. The prevalence of diabetes was 3.3% (95% CI 2.5 - 4.3%). Of the 52 participants who had diabetes, 25 (48%) were not aware that they had diabetes (new); and over half of the 27 who knew they had diabetes (52%) had high RBG > 11.1 mmol/L. Although the differences were not statistically significant, those who did not know they had diabetes were more likely to be younger than 50 years or older than 70 years, female and living in rural areas.

The prevalence of diabetes was highest in those aged 80 years and above (8.1% 95% CI 3.7-16.9), being 2.9% (95% CI 2.0-4.1%) among those aged 40-59 years, the economically active age group. There were no differences by gender or ethnic group (Table 2). The age-specific diabetes prevalence standardized to the 2012 population of Nigeria also showed increasing diabetes prevalence with increasing age above 40 years (Table 3). People with diabetes were more likely to live in urban areas, to be overweight or obese, literate, hypertensive (any stage) and blind (Table 4). In multivariate analysis, being aged 80 years and above, living in an urban location, and being overweight or obese remained independent risk factors.

**Table 2:** **Prevalence of diabetes**, **by socio**-**demographic and biophysical factors**, **in the subsample**

		Total	Diabetes	Prevalence		
		N	n	%	95% CI	P value
**Socio**-**demographic factors**						
Age group (years)	40 - 49	557	14	2.5	1.5-4.2	0.17
	50 - 59	412	14	3.4	2.0-5.6	
	60 - 69	346	11	3.2	1.7-5.9	
	70 - 79	206	7	3.4	1.6-6.9	
	80+	74	6	8.1	3.7-16.9	
		1595				
Gender	Female	852	23	2.7	1.8-4.0	0.19
	Male	743	29	3.9	2.7-5.7	
		1595				
Ethnic group	Fulani	83	2	2.4	0.6-8.9	0.87
	Hausa	384	11	2.9	1.6-5.0	
	Ibo	224	7	3.1	1.4-7.0	
	Yoruba	335	14	4.2	2.5-6.9	
	Others	562	18	3.2	2.0-5.1	
		1588*				
Literacy	Illiterate	894	21	2.4	1.5-3.6	0.02
	Literate	701	31	4.4	3.1-4.3	
		1595				
Location: residence	Rural	1240	31	2.5	1.8-3.5	0.001
	Urban	355	21	5.9	3.9-8.9	
Location: geopolitical zone	South East	192	5	2.6	1.0-7.0	0.90
	North West	432	12	2.8	1.7-4.7	
	North Central	267	8	3	1.4-6.2	
	South South	230	8	3.5	1.7-7.1	
	South West	343	13	3.8	2.3-6.2	
	North East	131	6	4.6	2.1-9.7	
**Biophysical factors**						
Blood pressure (mmHg)	Normal	878	21	2.4	1.6-3.7	0.03
	Hypertension (any)	710	31	4.4	3.1-6.1	
		1588*				
Blood pressure (mmHg)	Normal	878	21	2.4	1.6-3.7	0.13
	Hypertension stage 1	373	15	4	2.5-6.5	
	Hypertension stage 2	189	8	4.2	2.2-8.1	
	Hypertension stage 3	148	8	5.4	2.8-10.3	
		1588*				
Body mass index (kg/m^2^)	Underweight	187	3	1.6	0.5-4.9	<0.001
	Normal	968	20	2.1	1.3-3.2	
	Overweight	286	17	5.9	3.8-9.3	
	Obese	134	11	8.2	4.7-14.0	
		1575*				
Visual acuity	Not blind (>3/60)	1542	46	3	2.3-4.0	0.001
	Blind (<3/60)	53	6	11.3	5.1-23.2	
**Total**		**1595**	**52**	**3.3**	**2.5**-**4.3**	

*missing values excluded.

**Table 3:** **Age**-**standardized diabetes prevalence**

	Sub-sample		Prevalence of diabetes			
			**Crude rate**		**Age-adjusted rate** ^α^	
	**N**	**%**	**n**	**%**	**%**	**95% CI**
**Age group** **(years)**						
40-49	557	34.9	14	2.51	1.93	1.95-5.46
50-59	412	25.8	14	3.40	3.39	2.01-5.62
60-69	346	21.7	11	3.18	4.66	1.16-4.03
70-79	206	12.9	7	3.40	5.63	0.97-4.17
80+	74	4.6	6	8.11	6.17	4.86-22.22
Total	1595	100.0	52	3.26	3.25	2.50-4.30

^α^ = standardised to Nigeria 2012 population.

**Table 4:** **Univariate and multivariable analysis of risk factors for diabetes**

		Univariate analysis			Multivariate analysis		
		Odds ratio	95% CI	p-value	Odds ratio	95% CI	p-value
**Socio**-**demographic factors**							
Age group (years)	40 - 49	Reference			Reference		
	50 - 59	1.36	0.64-2.91	0.43	1.33	0.62-2.87	0.47
	60 – 69	1.27	0.55-2.96	0.58	1.59	0.67-3.76	0.29
	70 – 79	1.36	0.55-3.38	0.50	1.60	0.59-4.40	0.36
	80+	3.42	1.32-8.81	0.01	5.04	1.80-14.11	0.00
Gender	Female	Reference			Reference		
	Male	1.46	0.83-2.59	0.19	1.52	0.79-2.95	0.21
Ethnic group	Fulani	Reference			-	-	-
	Hausa	1.19	0.27-5.26	0.81			
	Ibo	1.31	0.26-6.54	0.74			
	Yoruba	1.77	0.41-7.69	0.45			
	Others	1.34	0.31-5.77	0.69			
Literacy	Illiterate	Reference			Reference		
	Literate	1.92	1.09-3.40	0.02	1.93	0.92-4.06	0.08
Location:	Rural	Reference			Reference		
Place of residence	Urban	2.45	1.39-4.32	0.00	1.87	1.01-3.47	0.05
Location:	South east	Reference			-	-	-
Geopolitical zone	North west	1.07	0.34-3.41	0.91			
	North central	1.16	0.32-4.14	0.82			
	South south	1.35	0.38-4.82	0.65			
	South west	1.47	0.47-4.64	0.51			
	North east	1.80	0.49-6.63	0.38			
**Biophysical factors**							
Blood pressure	Normal	Reference			-	-	-
	Hypertension (any)	1.86	1.06-3.29	0.03			
Blood pressure	Normal	Reference			Reference		
	Hypertension stage 1	1.71	0.88-3.31	0.11	1.37	0.70-2.70	0.36
	Hypertension stage 2	1.80	0.79-4.13	0.16	1.18	0.47-3.00	0.73
	Hypertension stage 3	2.33	1.00-5.44	0.05	1.38	0.53-3.62	0.51
Body mass index	Underweight	0.77	0.22-2.66	0.69	Reference	0.30-2.56	0.60
	Normal	Reference			1.00		
	Overweight	3.00	1.53-5.87	0.01	3.02	1.43-6.39	0.00
	Obese	4.24	2.02-8.89	0.00	4.43	1.82-10.78	0.00
Visual acuity	Not blind (VA >3/60)	Reference			Reference		
	Blind (VA <3/60)	4.15	1.69-10.22	0.00	3.20	1.10-9.30	0.03

*VA* = visual acuity.

### Prevalence of diabetic retinopathy and risk factors associated with diabetic retinopathy

In eight individuals the posterior pole could not be examined due to cataracts, corneal opacity or vitreous opacity (Table 5). The proportion of persons with diabetes with data on retinal findings who had any type of DR was 20.5% (9/44). Persons who knew they had diabetes had a higher rate of DR than persons newly diagnosed with diabetes: 25.0% (6/24) compared with 15.0% (3/20) respectively. Two thirds of the DR was sight-threatening, which affected 1 in 7 of persons with diabetes.

**Table 5:** **Retinal**/**macular findings in the most affected eye of participants with diabetes in the subsample**

	Known DM (n = 27)		New DM (n = 25)		Total (n = 52)	
	N	%	N	%	N	%
Unable to assess fundus (poor view)	3		5		8^#^	
**Data on retinal findings**	**24**	**100** **%**	**20**	**100** **%**	**44**	**100%**
Normal retina and macula	12		11		23	
Diabetic retinopathy:	6	25%	3	15%	9	20.5%
Non-proliferative	2		1		3	3
Proliferative	1		0		1	1*
Diabetic macular edema	3		2		5	5^@^
Other retinal/macular abnormality	6	25%	6	30%	12	27.3%

*DM* = diabetes mellitus; *NPDR* = non-proliferative diabetic retinopathy; *PDR* = proliferative diabetic retinopathy; *DME* = diabetic macular edema.

*NPDR in the other eye.

^@^3 had DME and NPDR in the same eye.

^#^Unable to view fundus in both eyes, or of other eye if one eye was normal: cataract (5), corneal opacity (1), vitreous haze (2).

Other retinal and/or macular pathology was detected in 12 (23.1%) of participants in the subsample with diabetes but who did not have DR (e.g. branch retinal vein occlusion).

### Risk factors for DR among people with diabetes in the subsample

In univariate analysis, the only factors associated with DR at the p ≤ 0.2 level were hypertension (any, and stages 2 and 3) and high RBG > 15.0 mmol/L (OR 9.0; 95% CI 0.89-91.43, p = 0.06). In multivariable analysis, high RBG > 15.0 mmol/L was the strongest predictor of DR but it did not reach statistical significance (OR 8.1; 95% CI 0.81-81.20). Confidence intervals were wide around all odds ratios due to the small sample size (data not shown).

### Visual impairment/blindness in diabetes and diabetic retinopathy

In the subsample 15/52 (28.8%) of those with diabetes were visually impaired. Uncorrected refractive error was the commonest cause (40%), followed by cataract and uncorrected aphakia (20% and 6% respectively). Other causes included optic atrophy, age related macular degeneration, corneal opacity and unexplained. People with diabetes were over three times more likely to be blind than those without diabetes (adjusted odds ratio 3.2, 95% CI 1.2-9.3) (Table 3).

### Types of diabetic retinopathy

In the subsample, six of the nine individuals with DR had STDR (one with PDR; five with DME). Among the 11,832 individuals not in the subsample 175 had a history of diabetes and 28 participants were identified with DR. Over half of the individuals with DR (16/28, 57.1%) did not know they had diabetes.

The types of retinopathy in the most affected eye in the 37 participants with DR (i.e. 9 in the subsample; 28 not in the subsample) were as follows: NPDR 27 (72.9%); PDR 4 (10.8%) and DME 19 (51.4%). Thus, STDR requiring assessment for treatment was identified in 23/37 (62.2%) participants with DR. Among the 37 participants with DR, 3 (8%) were blind, 27 (73.0%) had VI and 7 (19.0%) had normal vision. The commonest cause of VI was uncorrected refractive error; and DR was the cause of vision loss in two of the three blind individuals and in 4/27 with VI. All three who were blind knew they had diabetes and had advanced diabetic eye disease. One was a 42 year old man with a RBG >33.3 mmol/L. He had bilateral aphakia, optic atrophy and PDR with vitreous traction.

## Discussion

This study provides new and nationally representative data on the prevalence of diabetes mellitus and DR in Nigeria as well as socio-demographic and biophysical risk factors. Population-based nationally representative data are not available for most developing countries, particularly in Africa. An earlier Nigerian study reported the adjusted national standardised prevalence of diabetes to be 2.2% in all ages, which varied from 0.6% in a rural community to 7% in the urban population of Lagos, the former capital city [5]. Other studies in Nigeria involved small sample sizes in highly selected communities [8, 11, 29, 30] or urban populations (Port Harcourt [6] and Lagos [9]). The prevalence of diabetes reported in our study was not as high as that in Latinos (22.9%) [31], Saudi Arabia (29.7%; 95% CI 28.1-31.4%) [32] Mexico (21%, 95% CI 19.5-23.1%) [33], or in Asia (3.7% to 35%) [34–40] where a prevalence as high as 33.6% (95% CI 31.4-35.8) was reported in Singaporean Indians [38] and 35% (95% CI not reported) in the urban middle class population of Bangladesh [40]. The lower prevalence in Nigeria may reflect high levels of poverty and less exposure to known risk factors, shorter life expectancy, and importantly, poor control and high mortality among people with diabetes.

Many surveys report that a very high proportion of people with diabetes are unaware that they have the condition with most reporting that for every known person with diabetes there is at least one who is not diagnosed [6, 13, 41]. In our study, persons aged <50 or >70 years, females and those living in rural areas were more likely to have undiagnosed diabetes, indicating that these groups may be target groups for health education and diabetes testing.

As in our study, urban populations were at a greater risk of diabetes [5, 6], having two-fold greater risk [42]. It is postulated that urbanization is associated with changing lifestyles which lead to a high-calorie diet and obesity, and less physical activity. In Nigeria, women with a higher BMI have higher RBG levels [43] and type II diabetes [6] and our study also shows that being overweight/obese is an important risk factor for diabetes. Obesity is culturally acceptable and even desirable in many parts of Nigeria, and often seen as a marker of wealth and high standard of living. The same applies to having a car and not walking anywhere, or not working on the land or at home. Thus, behaviour and dietary change interventions may be very challenging and an area for further research in Nigeria.

The prevalence of hypertension, a major risk factor for diabetic retinopathy, is also high in Nigeria [14], being higher among those with diabetes.

The proportion of persons with diabetes who have DR varies in different populations, being high among adults in Mauritius (33%) [44], those aged 40 years and above in Latinos in Los Angeles, United States (47%) [31], Singaporean Malays (35%) [35], Singaporean Indians (30%) [38], the Handan Chinese (45%) [37] and in the those aged 50 years and above in Saudi Arabia (37%) [32] and Mexico (39%) [33]. However, reported rates were generally lower in studies undertaken in middle income countries , being similar to our study: i.e. 19% in Andra Pradesh, India [34], 18% in Chennai, India [36], 15% in Guangzhou, China [45] and 7.6% in Sao Paulo State, Brazil [46]. Reasons for the lower proportion of DR among persons with diabetes in Nigeria compared with high income settings are likely to reflect a combination of factors. Firstly, the onset of the epidemic of diabetes is recent and most people with diabetes would not have had the condition long enough to develop DR. Second, many of participants with diabetes had significant un-operated cataract which would underestimate DR. Third, poor control of diabetes, as demonstrated in our study, is likely to increase the risk of other complications such as cardiovascular disease, renal failure and infection [47, 48] and so increase the mortality rate. In our study, the proportion of DR that was sight threatening was high, possibly due to the high rates of uncontrolled diabetes and hypertension, but the sample size was small. Also, data on types of DR was derived from the whole study sample so the 28 cases of DR diagnosed in the non-normative sample would mainly have been detected as they had vision loss. In settings with highly efficient health systems and an educated population, rates of DR among people with diabetes can be very low. For example, in a study from Denmark only 7% of persons with diabetes had DR [49] and none was sight-threatening.

Previous reports on DR in Nigeria were hospital-based, and the findings are difficult to interpret due to variation in case mix, methods of detecting DR and in the classification systems used [50–59].

In our study, similar to reports from Los Angeles Latinos [31], Mexico [33] and Brazil [46] persons with diabetes had two-fold greater odds of being blind or visually impaired than persons without diabetes, with the commonest causes being cataract and uncorrected refractive error. In those studies, DR was also a major cause of blindness in people with diabetes [32, 33, 37]. However, in our study the contribution of DR to vision loss will have been underestimated, as the WHO method for assigning causes requires examiners to preferentially select treatable or preventable causes. For example, if an individual has diabetic macular edema and significant cataract, cataract should be selected as the cause as this is a readily reversible cause of vision loss.

Strengths of this study are that it included a nationally representative sample, and retinal images were read by an internationally recognised reading centre. The survey teams were highly experienced clinicians, and rigorous quality control mechanisms were in place.

However, several limitations in relation to diabetes and DR are acknowledged. First, for logistical reasons, RBG testing was not feasible on the whole sample, but was only performed on the subsample of participants. This meant that the CIs around the prevalence estimate are wide which limits the usefulness of estimates of magnitude. Second, RBG was used rather than fasting blood glucose as the latter was not feasible in the context of this very large survey. To make a definite diagnosis of diabetes, repeat and confirmatory tests need to be done. Thirdly, the diagnosis of diabetes was based on a RBG of >11.1 mmol/L performed by a glucose meter. RBG testing is not a widely accepted format for assessing prevalence of diabetes. It is a tool used mostly in clinical settings where other options are limited. We acknowledge that the accuracy and reproducibility of this method is poor but the results gave an estimate in a situation where there was dearth of data for prevalence of diabetes. Furthermore, the cut-off diagnostic value of >11.1 mmol/L would miss a number of people with altered glucose intolerance and diabetes. The aforementioned deficiencies could explain the numbers of diabetes being lower than might have been expected. The prevalence of diabetes presented here is thus, a minimum estimate. Another limitation was that diabetes was not classified as Type I or Type II, and information on the duration of diabetes was not sought. Due to lack of awareness of diabetes and inadequate primary and secondary health services in Nigeria, people present very late for a wide range of conditions, including diabetes. The year of diagnosis of diabetes would, therefore, markedly underestimate the duration of disease. The survey did not include individuals aged under 40 years of age, and diabetes may well be a problem in younger ages. Another limitation was that not all participants had dilated fundoscopy to detect DR and we might have missed some non-proliferative DR by direct fundoscopy in persons with normal visual acuity who did not have dilated retinal examination. In relation to risk factors for DR among people with diabetes, the power of the study to detect significant differences was low due to the small sample of participants with DR, and metabolic risk factors such as HbA1c were not assessed. Smoking was not explored as a risk factor, but cigarette smoking is unusual in Nigeria [60] and questioning about this habit would not have been acceptable to many participants. The limitations of this study underscore the need to have further studies to provide precise estimates on the prevalence of NCDs in Nigeria including diabetes mellitus and hypertension, in collaboration with physicians and NCD experts using accurate and acceptable guidelines for population-based diagnosis and surveys.

Diabetes and its complications are likely to have considerable economic consequences both for individuals, their families and society. In Nigeria, health insurance is not yet widely available, and government as well as private providers charge user fees for consultations. There are other out of pocket expenditures for medication, blood tests and other investigations, and for the management of complications. In our study one in every 35 adults of working age (40-59 years) had diabetes, which is likely to impact on economic productivity. Cost-effective and cost-saving interventions are urgently needed for the early detection and optimal management of diabetes in Nigeria [61] but it is recognised that there are scarcity of resources and numerous challenges to effective service delivery in Africa [62]. Poor awareness of the disease underscores the need for public health strategies for the diagnosis and treatment of diabetes, especially in high-risk groups. There is also a need for control of behavioural risk factors, such as diet and exercise, to curtail the burden of NCDs in Nigeria, through behaviour change interventions which are based on sound evidence. Other challenges in relation to DR include lack of equipment for diagnosis and treatment, an inadequately trained health workforce [62, 63], poor drug procurement and delivery mechanisms, low patient awareness and adherence to treatment, poor attendance at eye clinics despite referral and weak management information systems [55]. Using projections from the 2013 Diabetes Atlas, the number of people with diabetes in Nigeria is likely to double over the next two decades. There is a need for a national policy on screening for NCDs which is integrated at the primary level of care and which addresses all elements of the health system. Physicians involved in diabetic care, optometrists and other primary eye care workers should ensure an effective system for detecting DR among persons with diabetes with referral mechanisms for confirmatory diagnosis and treatment. It has been suggested that models of screening and treatment of DR which are being implemented in India can be adapted for sub-Saharan African countries [62]. Further research is required to determine the optimum modes of service delivery to prevent, detect and treat DR and how eye health systems can respond to the rapidly changing burden of disease.

## Conclusion

The study gives new epidemiological data for diabetes and DR in Nigeria. It is estimated that about 10% of people with diabetes aged ≥40 years in Nigeria may have sight-threatening diabetic retinopathy. The data will be relevant for development of health systems and services to respond to the growing burden of diabetes and its complications in sub-Saharan Africa.
